# Comparison of Brain Maturation among Species: An Example in Translational Research Suggesting the Possible Use of Bumetanide in Newborn

**DOI:** 10.3389/fneur.2013.00036

**Published:** 2013-04-15

**Authors:** Ronit Pressler, Stéphane Auvin

**Affiliations:** ^1^Institute of Child Health, University College London, London, UK; ^2^Department of Clinical Neurophysiology, Great Ormond Street Hospital, London, UK; ^3^INSERM, U676, Paris, France; ^4^INSERM UMR676, Université Paris Diderot, Paris, France; ^5^Service de Neurologie Pédiatrique, APHP, Hôpital Robert Debré, Paris, France

The therapeutic need for neurological diseases requires the pursuit of research in this area by the development of new models of brain diseases as well as preclinical drug studies. Among them, the development of medicines for newborns has been identified as an urgent need for both preclinical and clinical research (Silverstein et al., 2008). This would lead to more translational studies on the developing brain. However, there are certain risks involved in this translation from animal models to humans such as the effect on brain maturation, safety, and co-morbidity. Interpretation of results of preclinical drug studies requires a knowledge of brain maturation among species in particular when the efficacy or the safety of a drug may be different. Moreover, the risk benefit ratio of a drug in development should also be considered in the interpretation of translational studies.

The use of animal models in experimental studies has led to a dramatic increase in our knowledge. Rodents are the most frequently used species in both experimental and translational studies. In the field of developmental neuroscience several differences between human and laboratory rodent brain maturation are well recognized, but determining the exact equivalences in developmental milestones between species is a multidimensional task and a single answer is not always possible.

To put experimental data into clinical context, brain maturation among species is compared using various criteria such as cerebral growth, neurogenesis, synaptogenesis, and other variables (Table 1). These comparisons are done to propose translational research on the human developing brain. Using neurogenesis as a criterion, it has been shown that E18 and E21 rat brain match with week 8–9 and week 15–16 after fertilization in the human embryo, respectively (Bayer et al., 1993). But the timing of neurogenesis differs substantially across brain regions increasing the challenge to compare brain maturation between species. Moreover, most of the neuronal/astrocytic migration ends at 20 weeks of gestation (WG) in humans while this process is mainly observed between E19 and E21 in rats (Raedler et al., 1980). Other parameters such as functional measures could be used to evaluate postnatal development. As an example, the age at which the ability to move is achieved can be compared. Locomotion in the rat matures during the first few weeks after birth. Rat pups are able to ambulate through the use of their forelimbs, upper torso and head beginning around P3–P4. This ‘‘crawling’’ behavior peaks around P7 and disappears around P15. It is not until around P8–P10 that rat pups can stand with their abdomens completely off the floor. Around P12–P13, rat pups can walk while supporting their full weight, but the hindlimbs are typically rotated outward (Wood et al., 2003). In human infants, the last stage before walking around at 13 months of age typically involves intermittently placing one foot flat on the floor and creeping like a bear on hands and feet. Interestingly, an infant can bear his full weight (i.e., stand) while being held by her hands by 24–28 weeks and can walk while holding onto a piece of furniture by 48 weeks (Wood et al., 2003). Using several criteria, some authors have suggested a 12–13-day-old rat pup cerebral cortex can be compared to a term human newborn (Romijn et al., 1991).

**Table 1:** **Comparative development of the cortex between laboratory rodents and humans using various criteria**.

Parameters	Rodents	Humans	Reference
**Maximal growth velocity**	8–12 Postnatal days	2–3 Postnatal months	Gottlieb et al. (1977), Herschkowitz et al. (1997), Khazipov et al. (2001), Kretschmann et al. (1986)

**Neurogenesis**			
Birth	Mouse: E11–13 Rat: E12–15	GW5–6	Al-Ghoul and Miller (1989), Bayer et al. (1993), Del Rio et al. (2000), Kostovic and Rakic (1990), Price et al. (1997), Wood et al. (1992), Zeng et al. (2009)
Waiting	Mouse: E14–P0 Rat: E16–17	GW20–26	Catalano et al. (1991), Del Rio et al. (2000), Deng and Elberger (2003), Hevner (2000), Kostovic and Judas (2002)
Death (0–80%)	Mouse: E18-P21 Rat: E20–P30	GW34–41	Al-Ghoul and Miller (1989), Ferrer et al. (1990), Kostovic and Rakic (1990), Price et al. (1997), Wood et al. (1992)

**Neuronal migration**	Rats: mainly observed between E19 and E21 Mice: the preplate (PP) appears at E12. At E14, the intermediate zone is traversed by migrating neurons en route to the cortical plate (CP). At E16, the normal CP increases in thickness following the arrival of young neurons	This telencephalic – diencephalic migration occurs between 18 and 36 weeks PMA but mostly before 20 weeks of gestation	Bar et al. (2000), Gupta et al. (2005), Letinic and Kostovic (1997), Letinic and Rakic (2001), Raedler et al. (1980)

**Synaptogenesis**			
Duration of synaptogenesis	Rats: synaptogenesis continues for the first 3 weeks postnatally, peaking in the first 2 weeks	Synaptogenesis continues until approximately 3.5 years of age; the last structure to undergo synaptogenesis is the prefrontal cortex	Levitt (2003), Zagon and McLaughlin (1977)
First synapses	Rats: thalamocortical E17	Found at 9-10 weeks PMA in the cerebral cortex	Molliver et al. (1973), Zecevic et al. (1989)
Synaptic density and function	Rats: connections increase in neo-cortex from E16 to E21 Mice: P5-P6, 20% of fast-spiking neurons were electrically coupled; P15-P18, 42% of FS pairs had established electrical synapses	Synaptic density steadily increases with a rate of about 4% per week till 24-26 weeks PMA. Second increase of synaptic formation resulting in a 6-fold increase from 28 weeks PMA till the age at which the peak in synaptic density occurs	Huttenlocher and Dabholkar (1997), Kostovic and Jovanov-Milosevic (2006), Pangratz-Fuehrer and Hestrin (2011), Schlumpf et al. (1980), Zecevic (1998)

Reviewing these data on brain development, we think that the recent data published by Wang and Kriegstein (2011) don't provide any argument against the use of bumetanide in the human neonate. Wang and Kriegstein used different protocols of bumetanide administration in four different age groups showing that the early life blocking Na^+^-K^+^-2Cl^−^ co-transporter (NKCC1) with bumetanide disrupts the balance of excitatory and inhibitory synapses in the cortex of adult mice. These effects were shown in two of the treated groups. These groups received daily systemic injections of 0.2 mg/kg of bumetanide from the 15th day of gestation (E15) to the 7th postnatal day (P7) or from E17 to P7. Interestingly, no difference in the synapse excitability was observed when the mice became adult in the two other groups which were treated with bumetanide after birth (P0-P7 and P7-P14). The altered neurotransmission in adult mice that were exposed to bumetanide early in life (E15-P7 and E17-P7) was correlated with modifications of neuron morphology as well as behavior modifications. Behavior studies were conducted in adult mice from the E15-P7 group. Long-term consequences were observed such as developmental delay and impairment in sensorimotor gating. Similar findings were observed with other antiepileptic drugs (Forcelli et al., 2012a,b).

Bumetanide is a loop diuretic with a rapid onset and short duration of action blocking the renal NKCC1 co-transporter. Bumetanide is also able to block the neuronal NKCC1 co-transporter which is thought to be involved in the excitatory action of GABA in the immature brain (Ben-Ari and Holmes, 2006). The expression of NKCC1 in the developing brain starts embryonically in both human (gestational age of 20 weeks = GA20) and mouse (E12) but the peak of expression is prenatal in human (GA35) while it is postnatal in mice (P7) (Dzhala et al., 2005). Moreover, it has been shown that NKCC1 is increased by experimental hypoxic seizures in the developing brain (Cleary et al., 2013). It has been shown that bumetanide reduces neuronal firing in immature neurons using hippocampal slices (Dzhala et al., 2005) or intact hippocampus (Kilb et al., 2007). *In vivo* models studies have also shown the effect on seizure of bumetanide in both the kainate model (Dzhala et al., 2005) and the PTZ model in rat pups (Mares, 2009). Moreover, antiepileptogenic properties have been reported using the rapid kindling model in rat pups (Mazarati et al., 2009). Clinical trials in neonates are under way in the US (Harvard Medical School Boston, personal communication) and with the NEMO study about to start in Europe (EU FP7 funded collaborative project). A recent single case report in a 6-week-old baby has shown that bumetanide can reduce seizure duration and frequency with no clinical or metabolic side effects (Kahle et al., 2009).

However, Wang and Kriegstein (2011) concluded that “our data suggest caution for long-term use of bumetanide to treat neonatal seizures.” We think this is a rather inappropriate postulation as their data do not provide evidence against the use in term babies and it neglects the risk benefit ratio of antiepileptic drugs in this age group.

Although seizures in the immature brain do not cause the same neuronal damage as in the mature brain, it is now evident that seizures in early life interfere with the development of neuronal circuits thereby increasing the risk for subsequent seizures and adverse behavioral outcome (Holmes, 2005; Ben-Ari and Holmes, 2006). Furthermore, seizures can increase perinatal hypoxic brain damage (Miller et al., 2002) and can lead to later cognitive or neurological deficits (Ronen et al., 2007). The treatment of neonatal seizures has not changed over the last several decades and the first line antiepileptic drug for neonatal seizures remains phenobarbitone (Blume et al., 2009; Vento et al., 2010), despite the fact that phenobarbitone has show only limited efficacy in the neonatal period (Painter et al., 1999; Boylan et al., 2004). A Cochrane (Booth and Evans, 2004) showed that there is little evidence from randomized trials to support the use of any AED currently in use in the neonatal period. Furthermore most of the older antiepileptic drugs used, in particular phenobarbitone and phenytoin, increase neuronal apoptosis (Bittigau et al., 2002) and this may further increase neuronal insult. This effect has not been found with newer antiepileptic drugs such as levetiracetam (Kim et al., 2007).

In contrast to hundreds of studies in the adult population the efficacy and safety of newer antiepileptic drugs in newborn babies has not been adequately studied. This is due to the specific technical (biological sampling, assessment of drug effect), logistic (recruitment), and financial (cost too high for a small market) as well as legal (informed consent) difficulties (Chiron et al., 2008). This gives rise to an ethical predicament: babies should be protected from the potential risks of research but they may be harmed when given inadequately studied medicines and/or fail to benefit from more effective and less harmful newer agents. The lack of new antiepileptic drugs for neonatal seizures stands in contrast to a growing understanding of the different mechanisms that explain the susceptibility to seizures and the lack of response to conventional anticonvulsants (Ben-Ari and Holmes, 2006; Jensen, 2009). This knowledge presents important new possibilities for novel age-specific therapeutic strategies. One important example is the above mentioned finding that GABA has excitatory properties in the immature brain which may be susceptible to treatment with bumetanide (Dzhala et al., 2005). The above mentioned clinical trials on the efficacy of bumetanide are the first steps toward high standard, multicenter trials using innovative methods to improve outcome for neonates at risk for acute and long-term neurologic damage from neonatal seizures.

A large amount of data on the brain maturation in the various species show the complexities of developmental timing. It seems that laboratory rodent P0 matches with the antenatal or early premature human baby. Based on these, it is clear that the issue of bumetanide safety is still open. Inappropriate or premature caution against bumetanide trials could have far reaching consequences for a potentially beneficial drug for neonatal seizure while this drug has been widely used in neonates for non-seizure conditions.
